# Identification and Characterization of *Calonectria* Species Associated with Plant Diseases in Southern China

**DOI:** 10.3390/jof8070719

**Published:** 2022-07-09

**Authors:** Yunxia Zhang, Cantian Chen, Chao Chen, Jingwen Chen, Meimei Xiang, Dhanushka N. Wanasinghe, Tom Hsiang, Kevin D. Hyde, Ishara S. Manawasinghe

**Affiliations:** 1 Innovative Institute for Plant Health, Zhongkai University of Agriculture and Engineering, Guangzhou 510225, China; 18825187974@163.com (C.C.); chenchao_cc98@163.com (C.C.); jingwen075@163.com (J.C.); mm_xiang@163.com (M.X.); kdhyde3@gmail.com (K.D.H.); 2 Key Laboratory of Green Prevention and Control on Fruits and Vegetables in South China, Ministry of Agriculture and Rural Affairs, Guangzhou 510225, China; 3 Center for Mountain Futures, Kunming Institute of Botany, Chinese Academy of Sciences, Honghe 654400, China; dnadeeshan@gmail.com; 4 School of Environmental Sciences, University of Guelph, Guelph, ON N1G 2W1, Canada; thsiang@uoguelph.ca; 5 Center of Excellence in Fungal Research, Mae Fah Luang University, Chiang Rai 57100, Thailand

**Keywords:** new taxa, Nectriaceae, pairwise homoplasy index, polyphasic approaches, species complexes

## Abstract

*Calonectria* species are important plant pathogens on a wide range of hosts, causing significant losses to plant production worldwide. During our survey on phytopathogenic fungi from 2019 to 2021, diseased samples were collected from various hosts in Guangdong Province, China. In total, 16 *Calonectria* isolates were obtained from leaf spots, stem blights and root rots of species of *Arachis*, *Cassia*, *Callistemon*, *Eucalyptus*, *Heliconia*, *Melaleuca* and *Strelitzia* plants. Isolates were identified morphologically, and a multigene phylogenetic analysis of combined partial sequences of calmodulin (*cmd*A), translation elongation factor 1-alpha (*tef*1-α) and beta-tubulin (*β-tubulin*) was performed. These sixteen isolates were further identified as nine *Calonectria* species, with five new species: *Ca*. *cassiae*, *Ca*. *guangdongensis,* *Ca*. *melaleucae*, *Ca*. *shaoguanensis* and *Ca*. *strelitziae,* as well as four new records: Ca. *aconidialis* from *Arachis hypogaea*, Ca. *auriculiformis* from *Eucalyptus* sp., *Ca*. *eucalypti* from *Callistemon rigidus*, and *Ca*. *hongkongensis* from *Eucalyptus gunnii*. Moreover, we provide updated phylogenetic trees for four *Calonectria* species complexes viz. *Ca*. *colhounii*, *Ca*. *cylindrospora*, *Ca*. *kyotensis* and *Ca*. *reteaudii*. Our study is the first comprehensive study on *Calonectria* species associated with various hosts from subtropical regions in China. Results from the present study will be an addition to the biodiversity of microfungi in South China.

## 1. Introduction

*Calonectria* De Not. (Nectriaceae, Ascomycota) was introduced and typified by *Ca*. *daldiniana* De Not. which was later changed to *Ca*. *pyrochroa* (Desm.) Sacc. [1]. *Calonectria* species are characterized by producing a red perithecium, a vesicle with a long stipe produced from conidiophores, and cylindrical multi-septate conidia [2]. There are 126 species accepted in *Calonectria* [3,4,5,6] and 423 species epithets are listed in the Index Fungorum [7]. *Calonectria* species are widely distributed in tropical and subtropical regions [8]. They are important phytopathogens causing leaf spots, stem blights and root rots, leading to plant death on a wide range of hosts [9]. It has been reported that 335 plant species belonging to around 100 families including forest trees, crops and ornamental plants can be infected by *Calonectria* species [9]. Some prominent diseases caused by *Calonectria* species such as *Eucalyptus* leaf blight, *Calonectria* black rot on peanuts, and box blight can cause serious threats to plant production worldwide [10,11,12,13]. *Calonectria* species are regarded as the most important causal agents of leaf blight on *Eucalyptus* spp., which is the most devastating disease in Southeast Asia and South America [11,14,15]. Around 40 *Calonectria* species have been identified from *Eucalyptus* plantations and nurseries in Brazil and China [11,16]. *Calonectria ilicicola* Boedijn & Reitsma has been reported as the peanut black rot pathogen (CBR) in Africa, Asia, Australia and North America [13]. This disease incidence has reached 50% in some peanut plantations [10]. Box blight is caused by *Ca*. *pseudonaviculata* (Crous, J.Z. Groenew. & C.F. Hill) L. Lombard, M.J. Wingf. & Crous and *Ca*. *henricotiae* Gehesquière, Heungens & J.A. Crouch, and the disease is now found in over 20 countries throughout temperate regions and can cause severe losses [12].

Even though there are over 100 *Calonectria* species that have been identified, taxonomic studies on *Calonectria* species in China are comparatively few. There are 25 *Calonectria* species recorded from China, and 17 of them are associated with *Eucalyptus* [14,15,16,17]. In the tropics, the warm and humid climate is suitable for fungal infection and growth [9]. Thus, isolation and characterisation of tropical microfungi in China have a great significance to fungal biodiversity in this country. The objectives of this study were to isolate and identify *Calonectria* species associated with various plant diseases in Guangdong Province, China. In total, 11 isolates were collected and identified based on their morphology and the multi-gene molecular approaches. Seven species belonging to four species complexes were identified and characterised. Complete species descriptions and illustrations are provided for identified taxa. 

## 2. Materials and Methods

### 2.1. Sample Collection

**Figure 1:**
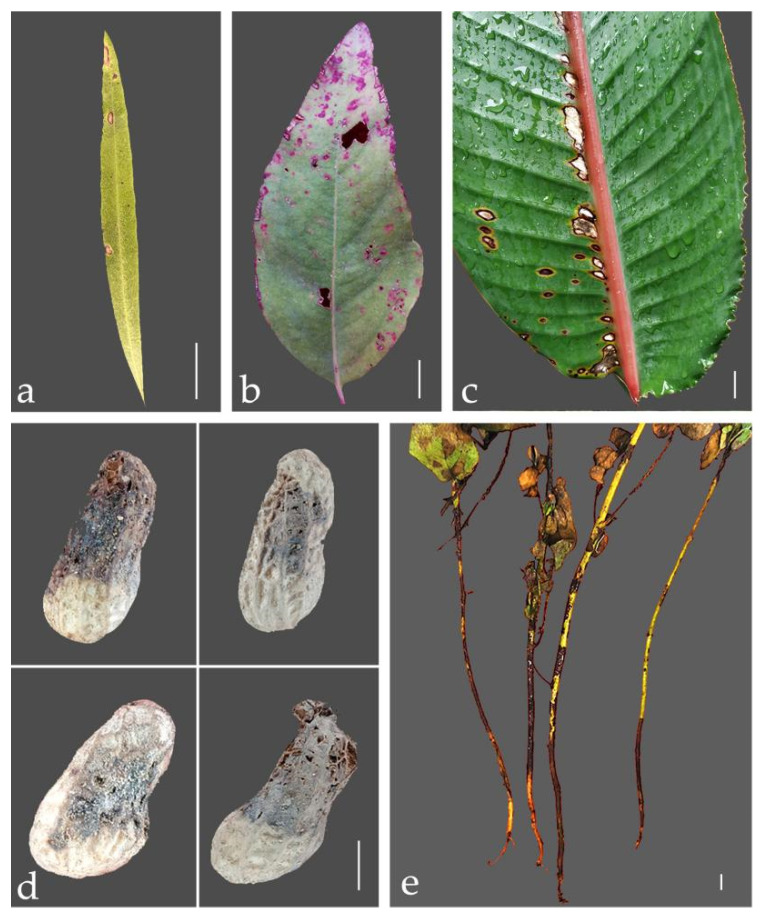
Field symptoms of diseased plants were collected from this study. (**a**) Leaf of *Callistemon rigidus*; (**b**) Leaf of *Eucalyptus* sp.; (**c**) Leaf of *Strelitzia reginae*; (**d**) Fruits of *Arachis hypogaea*; (**e**) Stems of *Cassia surattensis*; Scale bars: (**a**–**e**) = 1 cm.

Infected plant materials were collected from 2019 to 2021 in Guangdong, China from commercially grown plantations and nurseries. These samples included plants showing typical diseased symptoms of leaf spots, stem blights and root rots (Figure 1). In total, over 30 samples were collected from eight plant species, namely, *Arachis hypogaea* L., *Callistemon rigidus* R. Br., *Cassia surattensis* Burm., *Eucalyptus gunnii* Hook. f., *Eucalyptus* sp., *Heliconia metallica* Planch. et Linden ex Hook. f., *Melaleuca bracteata* F. Muell. and *Strelitzia reginae* Aiton. Photographs were taken and symptoms were recorded. Samples were placed loosely in bags with valves and kept cool and then brought back to the lab for further study.

### 2.2. Fungal Isolation

Diseased plant tissues were cut into small pieces (5 × 5 mm) which contained both healthy and diseased tissue. Then the surface was disinfected with 75% ethanol for 15–25 s and 2.5% NaClO for 40 s and rinsed in sterile water three times. After that, tissue pieces were dried on sterilized filter paper inside a laminar flow hood, and placed on potato dextrose agar (PDA) and incubated in the dark at 25 °C. Pure cultures were obtained after three serial transfers of hyphal tips. All cultures obtained in this study were deposited in the culture collection of Zhongkai University of Agriculture and Engineering (ZHKUCC). Herbarium materials (as dry cultures) were deposited in the herbarium of Zhongkai University of Agriculture and Engineering (ZHKU).

### 2.3. DNA Extraction and PCR Amplification

Genomic DNA was extracted using a DNA rapid Extraction Kit (Aidlab Biotechnologies Co., Ltd., Beijing, China) on 5-day-old cultures grown on PDA. Three loci, the calmodulin (*cmdA*), the translation elongation factor 1-alpha (*tef1-α*) and the beta-tubulin (*β-tubulin*) were amplified and sequenced using primers that were previously designed [18,19,20,21] (Table 1). PCR reaction mixtures consisted of 12.5 μL of 2 × Easy Taq PCR SuperMix (TransGen Biotech, Beijing, China), 2 μL DNA, 1 μL of each of the paired 5 μM primers, and ddH2O (8.5 μL). PCR reactions were conducted with an initial step of 95 °C for 3 min, followed by 35 cycles consisting of denaturation at 95 °C for 30 s, annealing from 53 to 55 °C (Table 1) for 30 s and extension at 72 °C for 1 min, and a final extension at 72 °C for 10 min. The reactions were performed in a C1000 TouchTM thermal cycler (Guangzhou Hongtu instrument Co., Ltd., Guangzhou, China). Amplified fragments were sequenced in both directions with forward and reverse primers by Guangzhou Tianyi Science and Technology Co., Ltd. (Guangzhou, China) and consensus sequences derived using BioEdit v.7.0.5.2 [22]. All sequence data generated in this study were submitted to NCBI GenBank (Supplementary Table S1).

### 2.4. Phylogenetic Analyses

For all isolates, the genus level was confirmed using the BLASTn tool (Basic Local Alignment Search Tool; https://blast.ncbi.nlm.nih.gov/Blast.cgi) at the National Center for Biotechnology Information (NCBI). To conduct phylogenetic analysis, sequences of *Calonectria* and other related species were obtained following Liu et al. [3]. Downloaded sequences were aligned with newly generated sequences using MAFFT v. 7 (https://mafft.cbrc.jp/alignment/server/). Sequences were improved manually when necessary, using BioEdit 7.0.5.2 [22]. Phylogenetic analyses were performed using concatenated datasets of *cmd*A, *tef*1-α and *β-tubulin* sequence data.

Phylogenetic relationships were inferred using maximum likelihood (ML) in RAxML [23] and Bayesian posterior probability analysis (BYPP) in MrBayes (v3.0b4) [24]. Maximum likelihood analyses and Bayesian analyses were accomplished on the CIPRES science gateway platform (http://www.phylo.org). The GTR + I + G evolution model was used with 1000 non-parametric bootstrapping iterations. The ML analysis was done with RAxML–HPC2 on XSEDE (8.2.8) [25,26] in the CIPRES Science Gateway platform [27]. For each phylogenetic tree, 1000 nonparametric bootstrapping iterations were used. Bayesian analyses were based on 2,000,000 generations, sampling every 100 generations, with four simultaneous Markov chains. Bayesian posterior probabilities were calculated after discarding a burn-in phase. The stability of the trees was evaluated by 1000 bootstrap replications. Descriptive statistics were calculated for the resulting trees.

### 2.5. Pairwise Homoplasy Index (PHI)

To confirm the species novelties, the pairwise homoplasy index (PHI index) was calculated. Here, the PHI index was calculated to determine species boundaries for the taxa with low tree values and significant evolution length. The PHI test was performed using SplitsTree4 v. 4 [28]. The concatenated three-locus dataset (*cmd*A + *tef*1-α + *β-tubulin*) was used for the analyses. The relationships between isolates belonging to this study and closely related taxa were visualized in split graphs with both the Log-Det transformation and split decomposition options.

### 2.6. Morphological Characterisation

Representative isolates incubated on carnation leaf agar (CLA) at 25 °C were used for morphological characterization [9]. Teleomorphic structures such as perithecia, asci and ascospores, and anamorphic structures such as the conidiophores, vesicles and conidia were photographed, and measurements were taken. The Cnoptec SZ650 (Chongqing Optec Instrument Co., Ltd., Chongqing, China) series stereomicroscope was used to observe macro-morphological characteristics. Micromorphological characters were observed using Nikon Eclipse 80i (Nikon, Tokyo, Japan). Morphological features including conidial length, width, and size were measured (at least 40 per isolate) using NISElements BR 3.2.

To observe culture characteristics, representative isolates were grown on malt extract agar (MEA) at 25 °C in the dark [9]. Colony diameters were examined after seven days with three replicate plates. Colony colors were recorded following the Rayner [29] color chart, and textures were observed daily until colonies covered the whole plate.

## 3. Results

### 3.1. Phylogenetic Analyses

**Figure 2:**
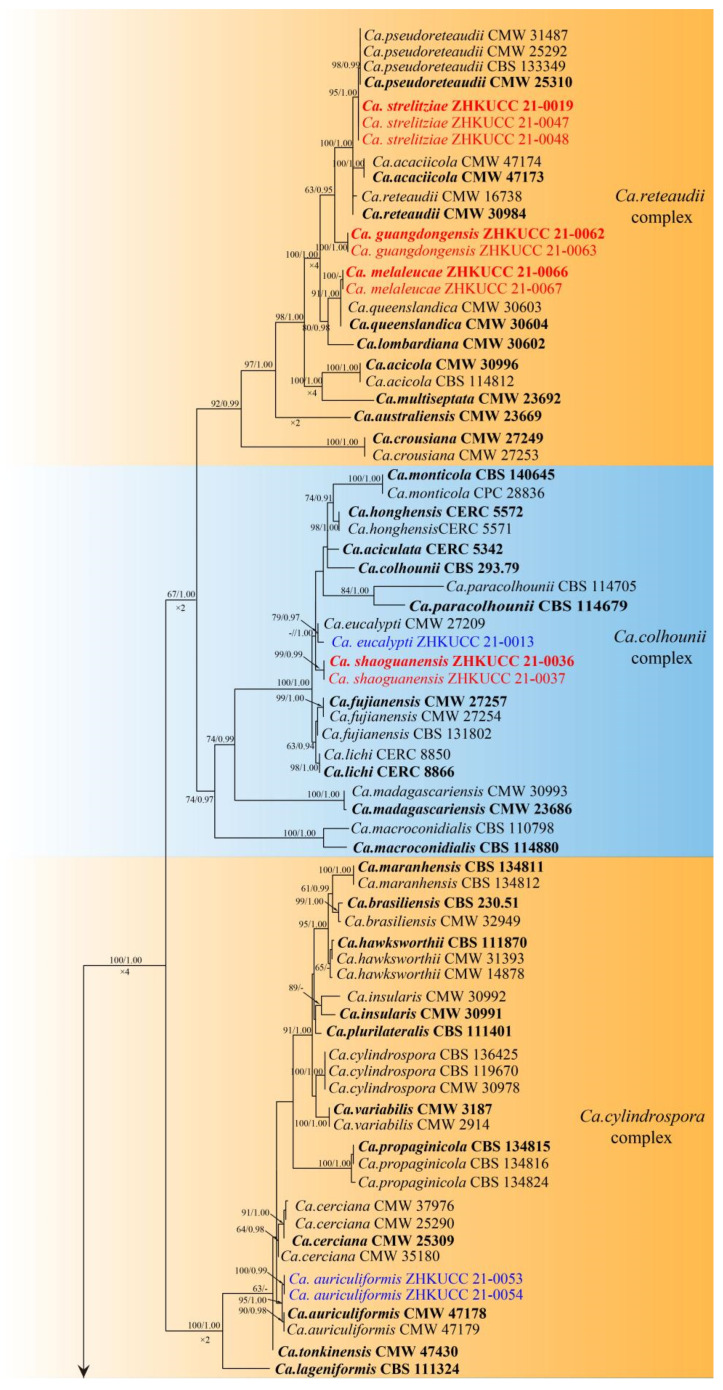

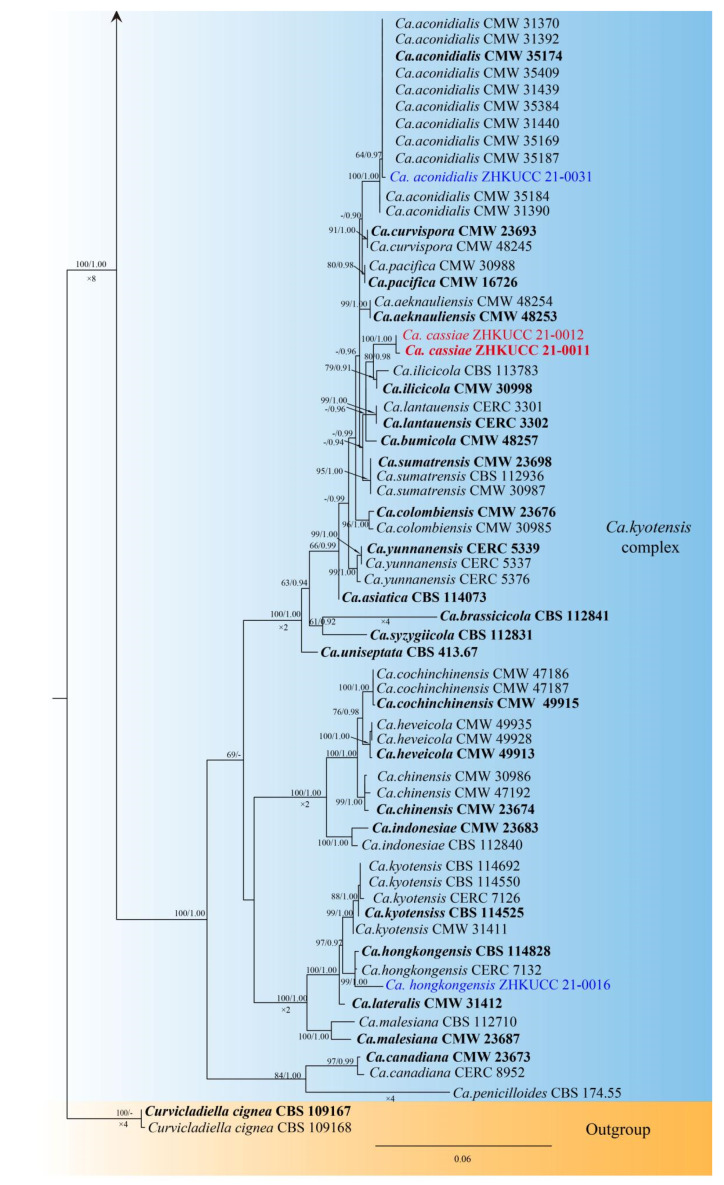
Phylogenetic tree of *Calonectria* species based on Maximum Likelihood (ML) analyses of the combined DNA dataset of *cmd*A, *tef*1-α and *β-tubulin* gene sequences. The ML bootstrap support values ≥60% and BYPP higher than 0.90 are indicated above the nodes and branches, and the ×2, ×4, and ×8 below the branches indicate that their lengths are compressed two-, four-, or eight-fold, respectively. The scale bar indicates 0.06 changes per site. Ex-type isolates of *Calonectria* species are marked in bold. Isolates for already known species in this study are in blue and novel taxa in this study are in red.

In total 16 *Calonectria* isolates were obtained. Phylogenetic analyses for *Calonectria* species were done using the concatenated dataset of *cmd*A, *tef* 1-α and *β-tubulin*. In total, sequences from 137 *Calonectria* strains including 16 strains from this study were used. *Curvicladiella cignea* (CBS 109167 and CBS 109168) was used as the outgroup. Tree topologies derived from the ML and BI analyse were congruent with each other; only the best scoring RAxML tree is presented (Figure 2). The best scoring RAxML tree had -14467.831159 as a final likelihood. The matrix had 998 distinct alignment patterns, with 14.59% of undetermined characters or gaps. Estimated base frequencies were as follows: A = 0.221391, C = 0.308101, G = 0.226807, T = 0.243702; substitution rates AC = 1.449245, AG = 3.672733, AT = 1.291386, CG = 0.870745, CT = 4.692338, GT = 1.000000; gamma distribution shape parameter α = 0.883863. The Bayesian analyses generated 4002 trees (average standard deviation of split frequencies: 0.015016) from which 3002 were sampled after 25% of the trees were discarded as burn-ins. The alignment contained a total of 988 unique site patterns.

In the phylogenetic trees, 16 isolates from this study were clustered in four species complexes in *Calonectria*: *Ca*. *colhounii, Ca*. *cylindrospora*, *Ca*. *kyotensis* and *Ca reteaudii*. These species formed nine distinct groups, from which four were grouped with already known species and five were novel species.

### 3.2. PHI Analyses

**Figure 3:**
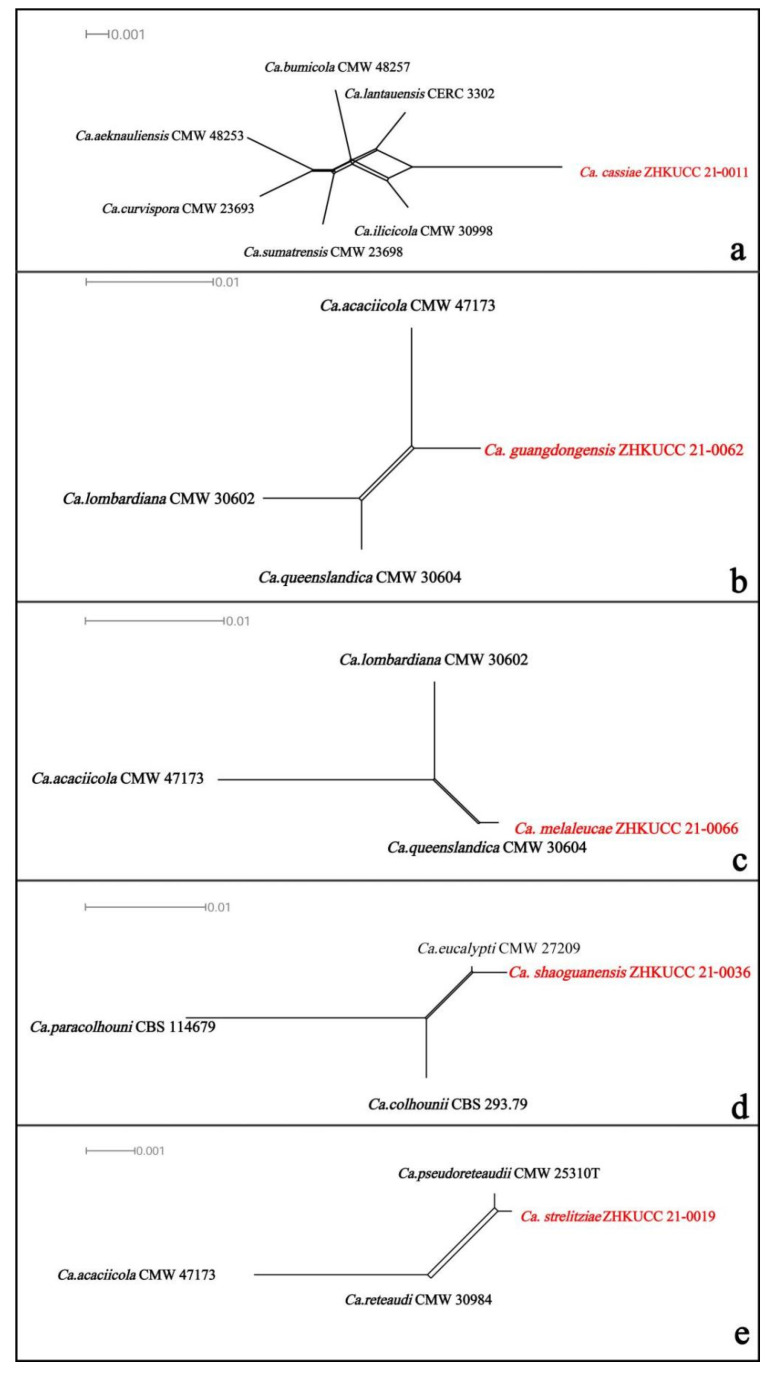
The results of the pairwise homoplasy index (PHI) test of five *Calonectria* new species and closely related species using both LogDet transformation and split decomposition. (**a**) *Ca*. *cassiae*; (**b**) *Ca*. *guangdongensis*; *(***c**) *Ca*. *melaleucae*; (**d**) *Ca*. *shaoguanensis*; (**e**) *Ca*. *strelitziae*.

To confirm the species novelties, the PHI index was calculated. The PHI analysis of five new species (*Ca. cassiae*, *Ca*. *guangdongensis*, *Ca*. *melaleucae*, *Ca*. *shaoguanensis* and *Ca*. *strelitziae*) and closely related taxa did not show significant recombination (the P-value was 1.0, 1.0, 0.06, 1.0 and 1.0, respectively) (Figure 3). This evidence provides support that the new taxa and closely species were different from each other. These results confirmed that these five taxa were different from the already known species of *Calonectria.*

### 3.3. Taxonomy

***Calonectria aconidialis*** L. Lombard, Crous & S.F. Chen bis, Stud. Mycol. 80: 162 (2015) Figure 4.

Index Fungorum number: IF 809043.

Associated with stem rot of *Arachis hypogaea.* Telemorph: *Perithecia* 290–600 × 240–480 µm, solitary, orange–red, subglobose, perithecial walls rough. *Asci* 110–140 × 15–20 µm, fusiform, eight-spored. *Ascospores* (32–)37–40(–46) × (5–)6–8(–9) µm ($\bar{x}$ = 38.5 × 6.5 µm, *n* = 50), hyaline, fusoid, straight to curved, 1-septate, sometimes constricted at septum. Anamorph: *Macroconidiophores* 15–25 × 4–6 μm, septate, hyaline. Primary branches of conidiogenous apparatus; secondary branches aseptate, 10–20 × 4–6 μm; tertiary branches 10–15 × 3–6 μm; each terminal branch producing two to four phialides, 10–15 × 3–6 μm. *Macroconidia* (44–)48–50(–55) × 4–7(–8) μm ($\bar{x}$ = 49.5 × 7 μm, *n* = 50), cylindrical, straight, 1-septate. Megaconidia and microconidia not observed.

Culture characteristics—Colonies on MEA fast growing at 25 °C growth rate 11 mm/d (*n* = 5), circular, producing abundant white aerial mycelium; reverse orange.

Material examined—China, Guangdong Province, Shaoguan City, *Arachis hypogaea* Linn., (*Fabaceae*), 27 September 2019, C.T. Chen, dried culture (ZHKU 21-0028), and living culture (ZHKUCC 21-0031).

Notes—One isolate from this study clustered with *Ca*. *aconidialis* in the multigene phylogeny with 64% ML and 0.97 BYPP values (Figure 2). Morphologically our isolate is similar to *Ca*. *aconidialis* as described by Lombard et al. [16] (Table 2). However, Mega, macro and microconidia were not observed in Lombard et al. [16], while our isolate produces macroconidia. To our knowledge, this is the first report of *Ca*. *aconidialis* from *Arachis hypogaea* [30].

***Calonectria auriculiformis*** N.Q. Pham, T.Q. Pham & M.J. Wingf., Mycologia 111(1): 85 (2019) Figure 5.

Index Fungorum number: IF 825527.

Associated with leaf spot of *Eucalyptus* sp. Telemorph: not observed. Anamorph: *Macroconidiophores* septate, hyaline. Primary branches of conidiogenous apparatus 10–30 × 3–10 μm; secondary branches aseptate, 10–25 × 3–8 μm; third branches 10–16 × 3–8 μm; tertiary branches 10–15 × 3–8 μm; each terminal branch producing two to four phialides, 10–20 × 3–6 μm. *Vesicles* 5–13 μm diamater, ellipsoidal to obpyriform. *Macroconidia* (38–)40–43(–47) × (3–)4–6 (–7) μm ($\bar{x}$ = 41.5 × 5 μm, *n* = 50), cylindrical, straight, 1-septate. Megaconidia and microconidia not observed.

Culture characteristics—Colonies on MEA fast growing at 25 °C, growth rate 9.5 mm/d (*n* = 5), circular, with regular margin, producing white aerial mycelium; reverse umber.

Material examined—China, Guangdong Province, Guangzhou City, *Eucalyptus* sp. L.Herit, (*Myrtaceae*), 11 July 2020, C.T. Chen, dried cultures (ZHKU 21-0050), and living culture (ZHKUCC 21-0053).

Notes—In the phylogenetic tree, two isolates from our study were closed to *Ca*. *auriculiformis* with 95% in ML, and 1.00 in BYPP support (Figure 2). Morphologically, our isolates are similar to *Ca*. *auriculiformis* described by Pham et al. [31] (Table 2).

**Figure 6:**
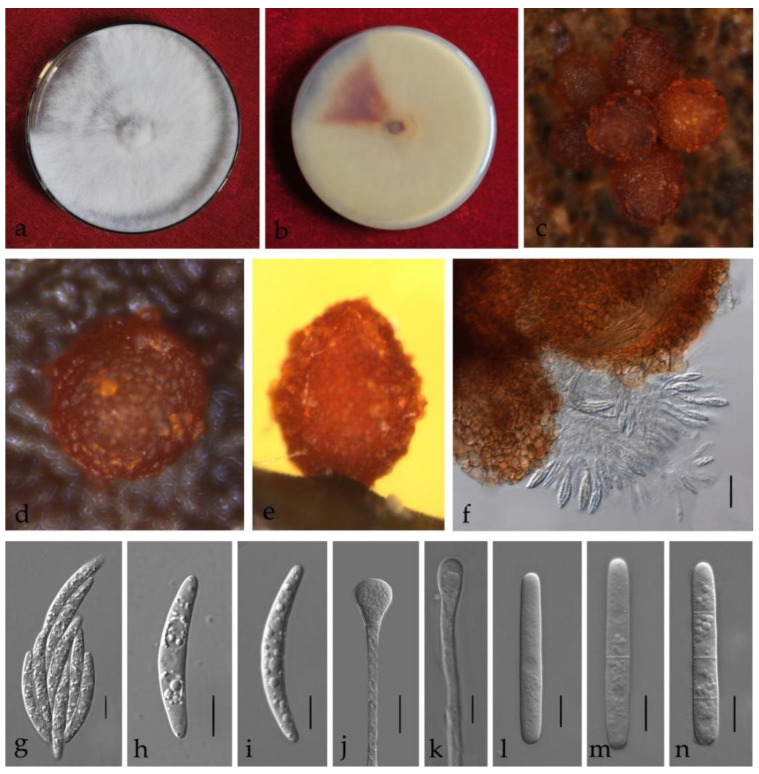
*Calonectria cassiae* (ZHKUCC 21-0011: Ex-type) (**a**,**b**) Culture characteristics on MEA after 7 days ((**a**), upper view; (**b**), reverse view); (**c**–**e**) Ascomata; (**f**,**g**) Asci; (**h**,**i**) Ascospores; (**j**,**k**) Vesicles; (**l**–**n**) Macroconidia; Scale bars: (**c**–**e**) = 100 μm; f = 50 μm; (**g**–**n**) = 10 μm.

***Calonectria cassiae*** Y. X. Zhang, C. T. Chen, Manawas., & M. M. Xiang, sp. nov. Figure 6.

Index Fungorum number: IF 553370.

Associated with stem rot of *Cassia surattensis.* Telemorph: *Perithecia* 400–700 × 350–550 µm, solitary, red–brown, subglobose to ovoid, perithecial walls rough. *Asci* fusiform with eight-spored. *Ascospores* 40–50 × 4–8 µm ($\bar{x}$ = 45 × 6 µm, *n* = 50), hyaline, fusoid, straight to slightly curved, 1-septate, constricted at septum. Anamorph: *Macroconidiophores* septate, hyaline. Primary branches of conidiogenous apparatus 15–25 × 4–6 μm; secondary branches aseptate, 10–20 × 4–6 μm; tertiary branches 10–15 × 3–6 μm; each terminal branch producing two to four phialides, 10–15 × 3–6 μm. *Vesicles* 7–12 μm diameter, sphaeropedunculate. *Macroconidia* 40–65 × 4–8 μm ($\bar{x}$ = 54 × 6 μm, *n* = 50), cylindrical, straight, 1–3-septate. Megaconidia and microconidia not observed.

Culture characteristics—Colonies on MEA fast growing at 25 °C, growth rate 11.2 mm/d (*n* = 5), circular, producing abundant white aerial mycelium; reverse red brown. 

Material examined—China, Guangdong Province, Guangzhou City, *Cassia surattensi* Burm. F., (*Fabaceae*), 30 March 2019, Y.X. Zhang, dried culture (ZHKU 21-0008, holotype), and living culture (ZHKUCC 21-0011, ex-type).

Notes—Two isolates obtained in this study developed a distinct sister clade to the *Ca*. *ilicicola* clade with 80% in ML and 0.98 in BYPP supports in phylogenetic analyses (Figure 2). Morphologically, the macroconidia of our isolates ($\bar{x}$ = 54 × 6 μm) are shorter than those of *Ca*. *ilicicola* ($\bar{x}$ = 62 × 6 μm) described by Lombard et al. [32] (Table 2), and microconidia were not observed in this study. In the PHI analysis of closely related taxa, there is no significant evidence of recombination among our isolates and other related species (*p* = 1.0). Therefore, we introduce *Ca*. *cassiae* from *Cassia surattensis* as a novel species based on phylogenetic analyses, morphological analyses and recombination analysis.

***Calonectria eucalypti*** L. Lombard, M.J. Wingf. & Crous, Stud. Mycol. 66: 47 (2010) Figure 7.

Index Fungorum number: IF 515530.

Associated with leaf spot of *Callistemon rigidus*. Telemorph: not observed. Anamorph: *Macroconidiophores* 15–45 × 4–8 μm, septate, hyaline. Primary branches of conidiogenous apparatus; secondary branches aseptate, 10–25 × 4–8 μm; tertiary branches 10–20 × 3–8 μm; each terminal branch producing two to six phialides, 8–20 × 3–6 μm. *Vesicles* 4–8 (–10) μm diameter, clavate to broadly clavate. *Macroconidia* (65–)70–80(–87) × (5–)6 μm ($\bar{x}$ = 75 × 6.5 μm, *n* = 50), cylindrical, straight, 1–3-septate. Megaconidia and microconidia not observed.

Culture characteristics—Colonies on MEA at 25 °C, growth rate 8.7 mm/d (*n* = 5), circular, producing abundant white aerial mycelium; reverse brown.

Material examined—China, Guangdong Province, Guangzhou City, *Callistemon rigidus* R. Br., (*Myrtaceae*), 10 November 2020, dried culture (ZHKU 21-0010), and living culture (ZHKUCC 21-0013).

Notes—In the phylogenetic tree, our isolates were grouped with *Ca*. *eucalypti* with 79% in ML and 0.97 in BYPP support (Figure 2). The macroconidia of the isolate belonging to this study ($\bar{x}$ = 75 × 6.5 μm) are similar to the size of the *Ca*. *eucalypti* ($\bar{x}$ = 72 × 6 μm) described by Lombard et al. [32]. In addition, the vesicle shape and the dimension of our isolate are similar to *Ca*. *eucalypti* [32] (Table 2). We introduce our isolates from *C. rigidus* as an anamorph of *Ca*. *eucalypti* based on phylogenetic analyses and asexual morphological characteristics. To our knowledge, this is the first report of *Ca*. *eucalypti* from *Callistemon rigidus* [30].

**Figure 8:**
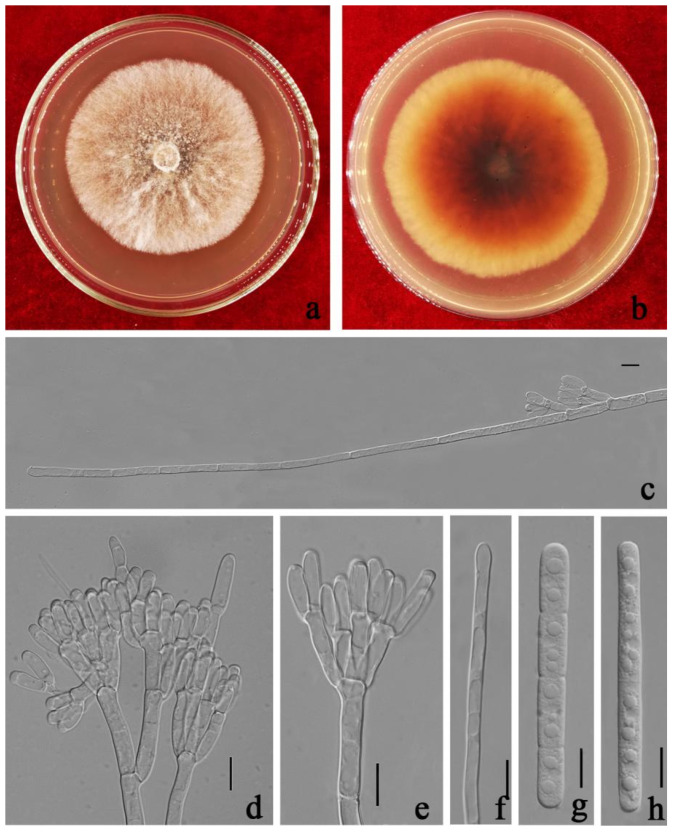
*Calonectriaguangdongensis* (ZHKUCC 21-0062: Ex–type) (**a**,**b**) Culture characteristics on MEA after 7 days (**a**), upper view; (**b**), reverse view); (**c**) Macroconidiophores; (**d**,**e**) Conidiogenous apparatus; (**f**) Vesicles; (**g**,**h**) Macroconidia; Scale bars: (**c**–**h**) = 10 μm.

***Calonectria guangdongensis*** Y. X. Zhang, C. T. Chen, Manawas., & M. M. Xiang, sp. nov. Figure 8

Index Fungorum number: IF 553380.

Etymology—Epithet refers to the Guangdong Province from where the type was collected.

Holotype—ZHKU 21-0059.

Associated with leaf spot of *Heliconia metallica*. Telemorph: not observed. Anamorph: *Macroconidiophores* septate, hyaline. Primary branches of conidiogenous apparatus 15–30 × 3–8 μm; secondary branches aseptate, 10–20 × 3–7 μm; tertiary branches aseptate, 10–15 × 3–6 μm; each terminal branch producing two to four phialides, 7–15 × 3–6 μm. *Vesicles* 3–7 μm diameter, narrowly clavate. *Macroconidia* 55–70 × 5–7(–9) μm ($\bar{x}$ = 64 × 6 μm, *n* = 50), cylindrical, straight, 1–3-septate. Megaconidia and microconidia not observed.

Culture characteristics—Colonies fast growing at 25 °C on MEA, growth rate 9.2 mm/d (*n* = 5), circular, producing abundant white aerial mycelium, reverse red brown.

Material examined—China, Guangdong Province, *Heliconia metallica* Planch. et Linden ex Hook. f., (*Musaceae*). 26 July 2020, Y.X. Zhang, dried cultures (ZHKU 21-0059, holotype), and living culture (ZHKUCC 21-0062, ex-type).

Notes—Our isolates from *Heliconia metallica* formed a distinct clade from the closely related taxa with 63% ML and 0.95 BYPP support. Morphologically our isolate differs from the other four closely related species by the size of the macroconidia. Our isolate (64 × 6 μm) developed macroconidia smaller than *Ca*. *acaciicola* N.Q. Pham, T.Q. Pham & M.J. Wingf. (94 × 7 μm), *Ca. pseudoreteaudii* L. Lombard, M.J. Wingf. & Crous (104 × 8 μm), *Ca. reteaudii* C. Booth (84 × 6.5 μm) and *Ca. strelitziae* (87 × 8 μm) [9,14,31] (Table 2). In the PHI analysis of closely related taxa, there is no significant evidence of recombination among our isolate and other related species (*p* = 1.0). Based on these polyphasic approaches, we introduce *Ca. guangdongensis* as a novel species from *H. metallica.*

***Calonectria hongkongensis*** Crous, Stud. Mycol. 50(2): 422 (2004) Figure 9.

Index Fungorum number: IF 500107.

Associated with leaf spot of *Eucalyptus gunnii*. Telemorph: not observed. Anamorph: *Macroconidiophores* septate, hyaline. Primary branches of conidiogenous apparatus 10–20 × 2–4 μm; secondary branches aseptate, 7–15 × 2–4 μm; tertiary branches 6–13 × 2–4 μm; each terminal branch producing two to three phialides, 5–10 × 1–3 μm. *Vesicles* 8–10 μm diameter, sphaeropedunculate to obpyriform. *Macroconidia* (38–)40–43(–46) × 4–6 μm ($\bar{x}$ = 42 × 5 μm, *n* = 50), cylindrical, straight, 1-septate. Megaconidia and microconidia not observed.

Culture characteristics—Colonies on MEA fast growing at 25 °C, growth rate 10.7 mm/d (*n* = 5), circular, with regular margin, producing abundant white aerial mycelium; reverse light-yellow to dark-brown.

Material examined—China, Guangdong Province, Guangzhou City, *Eucalyptus gunnii* Hook, (*Myrtaceae*), 11 July 2019, C.T. Chen and X. Sun, dried culture (ZHKU 21-0013), and living culture (ZHKUCC 21-0016).

Notes—In the phylogenetic analysis, a single isolate was obtained from *Eucalyptus gunnii* clustered with *Ca*. *hongkongensis* with 99% ML and 1.00 BYPP support (Figure 2). The anamorph of the isolate from this study is similar to *Ca*. *hongkongensis* described by Crous et al. [21] (Table 2). The telemorph was not observed in this study. To our knowledge, this is the first report of *Ca. hongkongensis* from *E. gunnii* [30].

**Figure 10:**
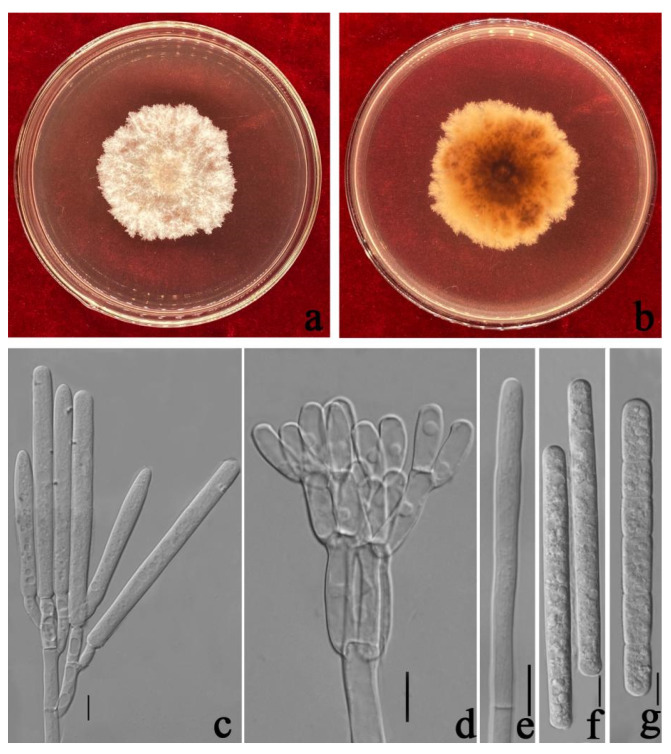
*Calonectriamelaleucae* (ZHKUCC 21-0066: Ex–type) (**a**,**b**) Culture characteristics on MEA after 7 days (**a**) upper view; (**b**) reverse view); (**c**,**d**) Conidiogenous apparatus; (**e**) Vesicle; (**f**,**g**) Macroconidia; Scale bars: (**c**–**g**) = 10 μm.

***Calonectria melaleucae*** Y. X. Zhang, C. T. Chen, Manawas., & M. M. Xiang, sp. nov. Figure 10.

Index Fungorum number: IF 553393.

Etymology—Epithet refers to the host (*Melaleuca bracteata*) from which the type was collected.

Holotype—ZHKU 21-0063.

Associated with leaf spot of *Melaleuca bracteata*. Telemorph: not observed. Anamorph: *Macroconidiophores* septate, hyaline. Primary branches of conidiogenous apparatus 15–35 × 3–7 μm; secondary branches aseptate, 15–25 × 3–6 μm; tertiary branches aseptate, 10–20 × 3–5 μm; each terminal branch producing two to four phialides, 10–15 × 3–5 μm. *Vesicles* 3–7 μm diameter, narrowly clavate. *Macroconidia* 80–95(–100) × (5–)7–10 μm ($\bar{x}$ = 88 × 8 μm, *n* = 50), cylindrical, straight, 3–5-septate. Megaconidia and microconidia not observed.

Culture characteristics—Colonies fast growing at 25 °C on MEA, growth rate 7.2 mm/d (*n* = 5), circular, producing abundant white aerial mycelium, reverse lightly red brown. 

Material examined—China, Guangdong Province, Guangzhou City, *Melaleuca bracteata* F. Muell., (*Myrtaceae*). 26 July 2020, Y.X. Zhang, dried cultures (ZHKU 21-0063 holotype), and living culture (ZHKUCC 21-0066, ex–type).

Notes—Our isolates from *Melaleuca bracteata* formed a distinct clade sister to *Ca. queenslandica* L. Lombard, M.J. Wingf. & Crous with 91% in ML and 1.00 in BYPP support. Our isolate differs from *Ca. queenslandica* by the size of the macroconidia [14]. Our isolate (88 × 8 μm) developed macroconidia larger than *Ca. queenslandica* (69 × 6 μm). In addition, our isolates are different from *Ca. queenslandica* in the number of macroconidia septa [3–5 vs. 4–6], as well as the size of vesicles (3–7 vs. 3–4 μm diameter) [14] (Table 2). In the PHI analysis of closely related taxa, there is no significant evidence of recombination among our isolate and other related species (*p* = 0.06). Based on these polyphasic approaches, we introduce *Ca. melaleucae* as a novel species from *M. bracteata.*

**Figure 11:**
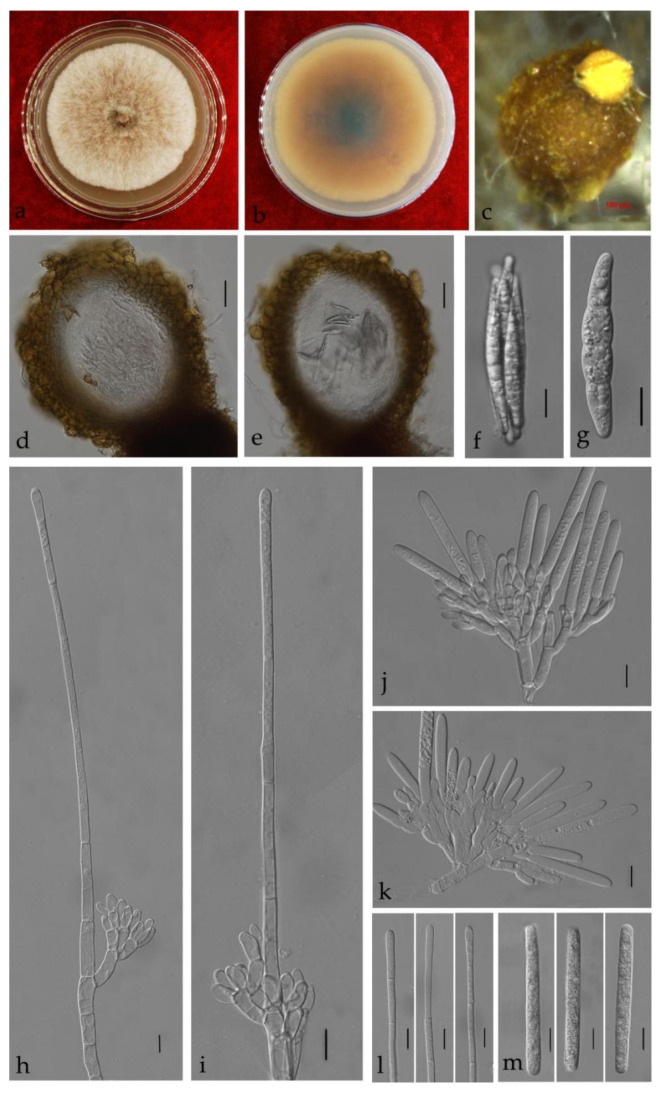
*Calonectria shaoguanensis* (ZHKUCC 21-0036: Ex-type) (**a**,**b**) Culture characteristics on MEA after 7 days (**a**) upper view; (**b**) reverse view); (**c**) Ascomata; (**d**,**e**) Vertical section through ascomata; (**f**) An ascus; (**g**) A ascospore; (**h**,**i**) Macroconidiophores; (**j**,**k**) Conidiogenous apparatus; (**l**) Vesicles; (**m**) Macroconidia; Scale bars: (**c**) = 100 μm; (**d**,**e**) = 50 μm; (**f**–**m**) = 10 μm.

***Calonectria shaoguanensis*** Y. X. Zhang, C. T. Chen, Manawas., & M. M. Xiang, sp. nov. Figure 11.

Index Fungorum number: IF 555217.

Etymology—Epithet refers to the Shaoguan city from where the type was collected.

Holotype—ZHKU 21-0033.

Associated with leaf spot of *Callistemon rigidus*. Telemorph: *Perithecia* 250–500 × 200–380 µm, solitary, yellow, subglobose to ovoid, and rough perithecial walls. *Asci* fusiform, eight-spored. *Ascospores* (45–)50–70 × (3–)4–8(–9) µm ($\bar{x}$ = 56.5 × 6.5 µm, *n* = 50), hyaline, fusiform, straight to curved, 1-septate, sometimes constricted at septum. Anamorph: *Macroconidiophores* septate, hyaline. Primary branches of conidiogenous apparatus 10–25 × 3–8 μm; secondary branches aseptate, 10–18 × 3–7 μm; tertiary branches aseptate, 8–15 × 3–6 μm; each terminal branch producing two to four phialides, 7–12 × 2–5 μm. *Vesicles* (2–)4–7 μm diameter, narrowly clavate. *Macroconidia* (55–)60–70(–75) × (4–)5–8 μm ($\bar{x}$ = 65 × 6.5 μm, *n* = 50), cylindrical, straight, 1–3-septate. Megaconidia and microconidia not observed.

Culture characteristics—Colonies fast growing at 25 °C on MEA, growth rate 10 mm/d (*n* = 5), circular, producing abundant white aerial mycelium, reverse red brown.

Material examined—China, Guangdong Province, Shaoguan City, *Callistemon rigidus* R. Br., (*Myrtaceae*). 21 June 2020, Y.X. Zhang, dried cultures (ZHKU 21-0033 holotype), and living culture (ZHKUCC 21-0036 ex-type).

Notes—Our isolates from *Callistemon rigidus* formed a distinct clade from the other six closely related species with less than 60% ML and 1.00 BYPP support (Figure 2). Morphologically our isolate differs from *Ca. eucalypti* by the size of the macroconidia [32]. Our isolate (65 × 6.5 μm) developed macroconidia shorter than the two isolates of *Ca. eucalypti* described by Lombard et al. (72 × 6 μm) [32] and in this study (75 × 6.5 μm). In the PHI analysis of closely related taxa, there is no significant evidence of recombination among our isolate and other related species (*p* = 1.0). Based on these polyphasic approaches we introduce *Ca. shaoguanensis* as a novel species from *C. rigidus.*

**Figure 12:**
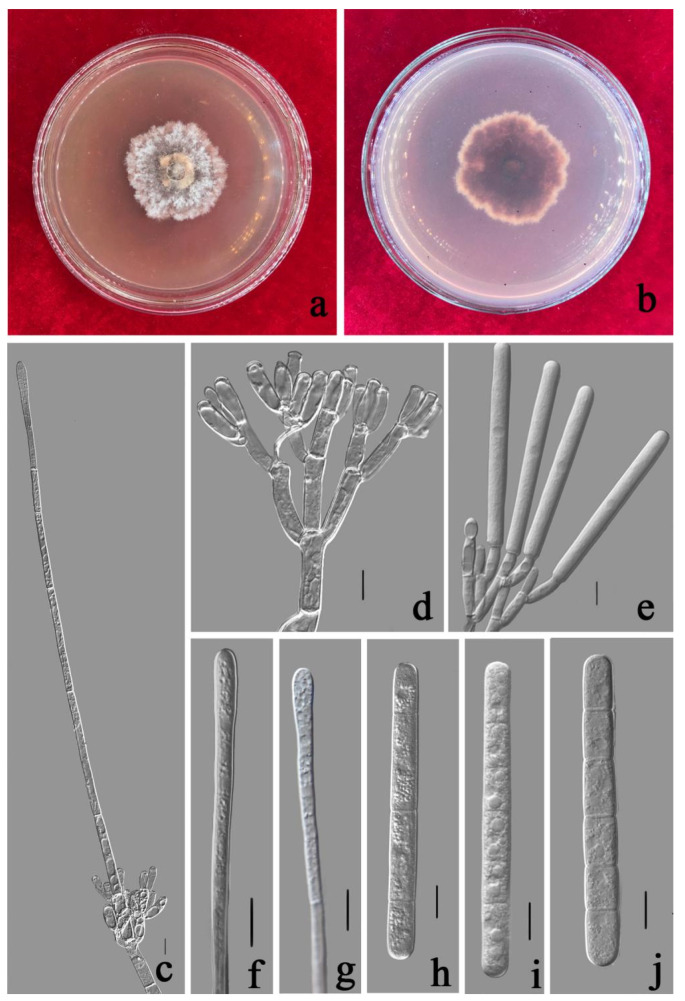
*Calonectria strelitziae* (ZHKUCC 21-0019: Ex-type) (**a**,**b**) Culture characteristics on MEA after 7 days (**a**) upper view; (**b**) reverse view); (**c**) Macroconidiophores; (**d**,**e**) Conidiogenous apparatus; (**f**,**g**) Vesicles; (**h**–**j**) Macroconidia; Scale bars: (**c**–**j**) = 10 μm.

***Calonectria strelitziae*** Y. X. Zhang, C. T. Chen, Manawas., & M. M. Xiang, sp. nov. Figure 12.

Index Fungorum number: IF 553395.

Etymology—Epithet refers to the host (*Strelitzia reginae*) from which the type was collected.

Holotype—ZHKU 21-0016.

Associated with leaf spot of *Strelitzia reginae*. Telemorph: not observed. Anamorph: *Macroconidiophores* septate, hyaline. Primary branches of conidiogenous apparatus 15–30 × 3–9 μm; secondary branches aseptate, 10–25 × 3–9 μm; tertiary branches aseptate, 10–25 × 4–8 μm; each terminal branch producing two to four phialides, 10–20 × 3–8 μm. *Vesicles* 3–8 μm diameter, narrowly clavate. *Macroconidia* (65–)80–95(–115) × (4–)6–10(–12) μm ($\bar{x}$ = 87 × 8 μm, *n* = 50), cylindrical, straight, 3–5-septate. Microconidia 45–55 × 4.7–5.5 ($\bar{x}$ = 49 × 5.1 μm, *n* = 9), cylindrical, 1-septate. Megaconidia not observed.

Culture characteristics—Colonies fast growing at 25 °C on MEA, growth rate 5.4 mm/d (*n* = 5), circular, with irregular edge, producing white aerial mycelium, reverse red brown. 

Material examined—China, Guangdong Province, Guangzhou City, *Strelitzia reginae* Aiton, (*Musaceae*). 11 July 2020, Y.X. Zhang and C.T. Chen, dried cultures (ZHKU 21-0016, holotype), and living culture (ZHKUCC 21-0019, ex-type).

Notes—Our isolates from *Strelitzia reginae* formed a single lineage sister to *Ca. pseudoreteaudii* with 95% in ML, and 1.00 in BYPP support (Figure 2). Morphologically our isolate differs from *Ca. pseudoreteaudii* by the shorter macroconidia (87 × 8 μm vs. 104 × 8 μm) and smaller microconidia (49 × 5 μm vs. 44 × 4 μm) [14] (Table 2). In the PHI analysis of closely related taxa, there is no significant evidence of recombination among our isolate and other related species (*p* = 1.0). Based on these polyphasic approaches, we introduce *Ca. strelitziae* as a novel species from *S. reginae.*

**Table 2:** Morphological comparison of *Calonectria* species obtained in this study with their relatives.

Complex	Species	Ascospores		Macroconidia		Vesicle		References
		Size/μm	Septa	Size/μm	Septa	Shape	Diam/μm	
*Ca. colhounii*	*Ca. eucalypti*	(25–)30–36(–56) × (3–)5–6(–8) $\bar{x}$ = 33 × 6	(1–)3	(66–)69–75(–80) × (5–)6 $\bar{x}$ = 72 × 6	3	broad clavate	4–6	[32]
	*Ca. eucalypti*	/	/	(65–)70–80(–87) × (5–)6 $\bar{x}$ = 75 × 6.5	1–3	Clavate to broad clavate	4–10	(This study)
	*Ca. shaoguanensis*	(45–)50–70 × (3–)4–8(–9) $\bar{x}$ = 56.5 × 6.5	1	(55–)60–70(–75) × (4–)5–8 $\bar{x}$ = 65 × 6.5	1–3	narrowly clavate	(2–)4–7	(This study)
*Ca. kyotensis*	*Ca. ilicicola*	$\bar{x}$ = 45 × 6	1	$\bar{x}$ = 62 × 6	1(–3)	sphaeropedunculate	8–12	[32]
	*Ca. cassiae*	40–50 × 4–8 $\bar{x}$ = 45 × 6	1	40–65 × 4–8 $\bar{x}$ = 54 × 6	1–3	sphaeropedunculate	8–12	(This study)
*Ca. reteaudii*	*Ca. strelitziae*	/	/	(65–)80–95(–115) × (4–)6–10(–12) $\bar{x}$ = 87 × 8	3–5	narrowly clavate to broad clavate	3–8	(This study)
	*Ca. pseudoreteaudii*	/	/	(88–)96–112(–119) × 7–9(–10) $\bar{x}$ = 104 × 8	5–8	narrowly clavate	3–5	[14]
	*Ca. acaciicola*	/	/	(85–)90–98(–105) × (6–)6.5–7.5 $\bar{x}$ = 94 × 7	5	narrowly clavate	4–7	[31]
	*Ca. reteaudii*	/	/	(50–)75–95(–120) × (5–)6–7 $\bar{x}$ = 84 × 6.5	(1–) 5(–6)	clavate	3–5(–6)	[9]
	*Ca. guangdongensis*	/	/	55–70 × 5–7(–9) $\bar{x}$ = 64 × 6	1–3	narrowly clavate	3–7	(This study)
	*Ca. melaleucae*	/	/	80–95(–100) × (5–)7–10 $\bar{x}$ = 88 × 8	3–5	narrowly clavate	3–7	(This study)
	*Ca. queenslandica*	/	/	(61–)65–73(–78) × (4–)5–6(–7) $\bar{x}$ = 69 × 6	4–6	narrowly clavate	3–4	[14]
	*Ca. lombardiana*	/	/	(64–)74–86(–98) × (5–)5.5–6.5(–7.5) $\bar{x}$ = 80 × 6	5	narrowly clavate	2–4	[3]

## 4. Discussion

Species identification is the most important step in plant pathology to develop control measures [33,34]. Thus, proper species identification is necessary following polyphasic approaches [33,34,35,36]. *Calonectria* represents one of the most important phytopathogenic genera in Nectriaceae [37]. In the present study, we isolated and identified seven *Calonectria* species associated with various leaf and root diseases from eight different hosts in China. These species were identified as five novel species: *Ca. cassiae*, *Ca. guangdongensis, Ca. melaleucae*, *Ca. shaoguanensis* and *Ca. strelitziae,* as well as four new host records: *Ca. aconidialis* from *Arachis hypogaea*, *Ca. auriculiformis* from *Eucalyptus* sp., *Ca. eucalypti* from *Callistemon rigidus*, and *Ca. hongkongensis* from *Eucalyptus gunnii*. *Calonectria* species belong to 11 species complexes [3] revealing that the diversity among these species is considerably high. Thus, the use of either morphology or phylogeny alone is difficult to provide strong support to delineate the species [11,16]. Therefore, in the present study, we used polyphasic approaches including morphology, phylogeny and recombination analysis to introduce novel species.

In this study, four *Calonectria* species were identified as new host records. *Calonectria eucalypti* were isolated from *Eucalyptus grandis* leaf from Sumatera Utara [32]. Here *Ca. eucalypti* is reported to be associated with leaf spots on *Callistemon rigidus*. Previous studies have mentioned that *Ca. colhounii* Peerally, *Ca. kyotensis* Terash. and *Ca. pteridis* Crous, M.J. Wingf. & Alfenas are associated with *C. rigidus* [9], and our collection, *Ca. eucalypti* will be a new addition. *Calonectria aconidialis* was introduced as a soil fungus from *Eucalyptus* plantation [16]. In the present study, *Ca. aconidialis* was isolated from rotted *A. hypogaea* pods. There are no host records for *Ca. aconidialis* worldwide [30]. Therefore, our study provides a novel ecological niche for *Ca. aconidialis*. However, further studies are required to understand the host shifting and pathogenicity mechanisms of this fungus.

*Calonectria hongkongensis* was described by Crous et al. from the soil in Hong Kong [21]. Up to now, 15 *Calonectria* species, including *Ca. hongkongensis,* have been isolated from *Eucalyptus* plants or plantation soils in China [17]. This species was only isolated from *Eucalyptus* plantation soil and was not identified as a pathogen on *Eucalyptus* leaves [16,17,38]. Therefore, *Ca. hongkongensis* is considered a soil-borne species that inhabits the soil [17]. This is the first report of *Ca. hongkongensis* being isolated from diseased *E. gunnii* roots, reflecting that this species can be a potential soil-borne pathogen in *Eucalyptus* plantations. *Calonectria auriculiformis* was described by Pham et al. from *Acacia auriculiformis* plantation soil in Vietnam [31]. Later it was isolated from soil in *E. urophylla* hybrid plantations in China [17]. Similar to *Ca. hongkongensis, Ca. auriculiformis* has not been reported as a plant pathogen in previous studies. In the present study, we isolated *Ca. auriculiformis* from diseased leaves of *Eucalyptus* in Guangdong, China. However, further assays including controlled inoculation studies are required to confirm the pathogenicity of these isolated taxa.

*Calonectria shaoguanensis* was introduced as a new species, while adding one more species to the *Ca. colhounii* complex. This species complex includes 11 species: *Ca. aciculata* Jie Q. Li, Q.L. Liu & S.F. Chen, *Ca. colhounii*, *Ca. eucalypti*, *Ca. fujianensis* S.F. Chen, L. Lombard, M.J. Wingf. & X.D. Zhou, *Ca. honghensis* Jie Q. Li, Q.L. Liu & S.F. Chen*, Ca. indusiata* (Seaver) Crous, *Ca. lichi* Q.L. Liu & S.F. Chen*, Ca. macroconidialis* (Crous, M.J. Wingf. & Alfenas) Crous*, Ca. madagascariensis* Crous*, Ca. monticola* L. Lombard & Crous and *Ca. paracolhounii* L. Lombard & Crous [3]. *Calonectria shaoguanensis* can be distinguished from its closely related species by its macroconidial dimensions and by the shape of vesicles as well as by the number of ascosporous septa and macroconidial septa [32]. This species was isolated from *Callistemon rigidus* leaf spot. There have been three *Calonectria* species found on *C. rigidus* previously [30].

Three novel species, *Ca. guangdongensis, Ca. melaleucae* and *Ca. strelitziae* were added into the *Ca. reteaudii* complex, which accommodates nine species [3]. Species in the *Ca. reteaudii* complex have narrowly clavate vesicles, orange to red–brown perithecia and generally >3-septate macroconidia [3]. These three new species differ from closely related taxa by the size of macroconidia, *Ca. guangdongensis* and *Ca. strelitziae* developed shorter macroconidia, *Ca. melaleucae* formed larger macroconidia [3,9,14,31]. In addition, the macroconidia septa number of *Ca. guangdongensis* was one to three, similar to that of *Ca. crousiana* and *Ca. australiensis*, while distinct from most species having over 3-septate macroconidia in the *Ca. reteaudii* complex [3]. *Calonectria cassiae* was introduced as a new species while adding one more species to the *Ca. kyotensis* complex, which is a larger complex with twenty-four species [3]. The novel species differ from closely related species *Ca. ilicicola* by producing shorter macroconidia [32]. Until now, there are fifteen fungal species associated with *Cassia surattensis*, while there are no previous records from *Calonectria* species [30]. This is the first report of *Calonectria* species being associated with *C. surattensis* stem and root rots.

Overall, in the present study, novel *Calonectria* species and new host records were identified. Here, these taxa were isolated from diseased plant tissues including leaf spots, stem and root rots. However, the pathogenicity of these isolates was not confirmed in this study. The identification and characterization of novel taxa from southern China contributes to the knowledge of the biodiversity resources in tropical regions. Moreover, this study adds information on the taxonomy and diversity of *Calonectria* species in China. Future studies are required to confirm the pathogenicity of these isolated species on different plant hosts, and to examine their biology and ecology.

## Figures and Tables

**Figure 4:**
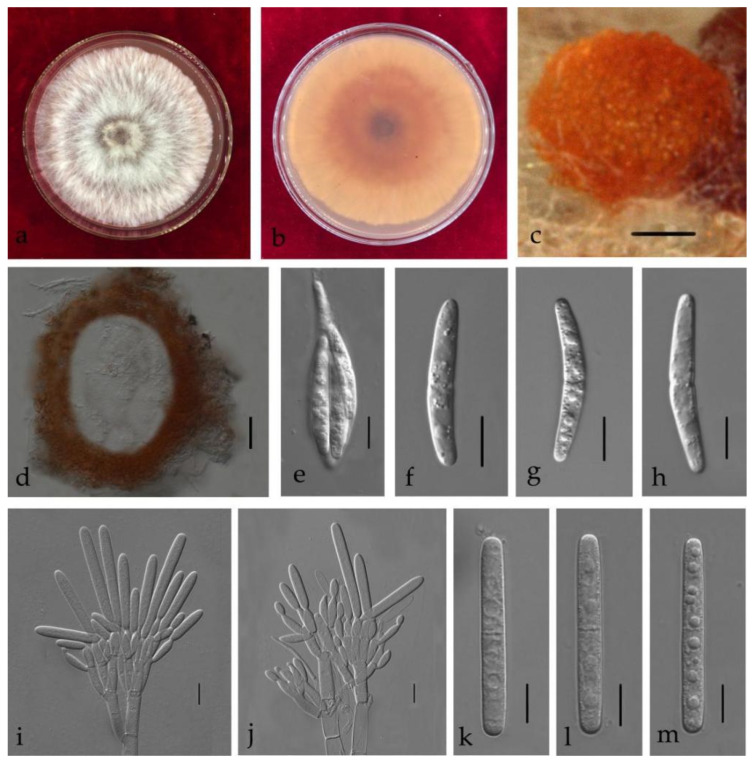
*Calonectria aconidialis* (ZHKUCC 21-0031: New host record) (**a**,**b**) Culture characteristics on MEA after 7 days ((**a**), upper view; (**b**), reverse view); (**c**) Ascomata; (**d**) Vertical section through an ascoma; (**e**) An ascus; (**f**–**h**) Ascospores; (**i**,**j**) Conidiogenous apparatus; (**k**–**m**) Macroconidia; Scale bars: c = 100 μm; d = 50 μm; (**e**–**m**) = 10 μm.

**Figure 5:**
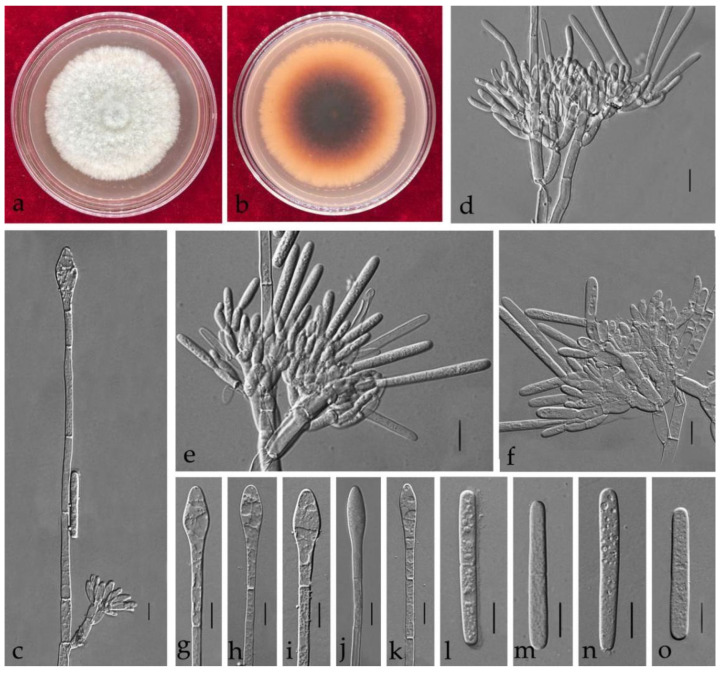
*Calonectria auriculiformis* (ZHKUCC 21-0053: New host record) (**a**,**b**) Culture characteristics on MEA after 7 days (**a**) upper view; (**b**) reverse view); (**c**) Macroconidiophores; (**d**–**f**) Conidiogenous apparatus; (**g**–**k**) Vesicles; (**l**–**o**) Macroconidia; Scale bars: (**c**–**o**) = 10 μm.

**Figure 7:**
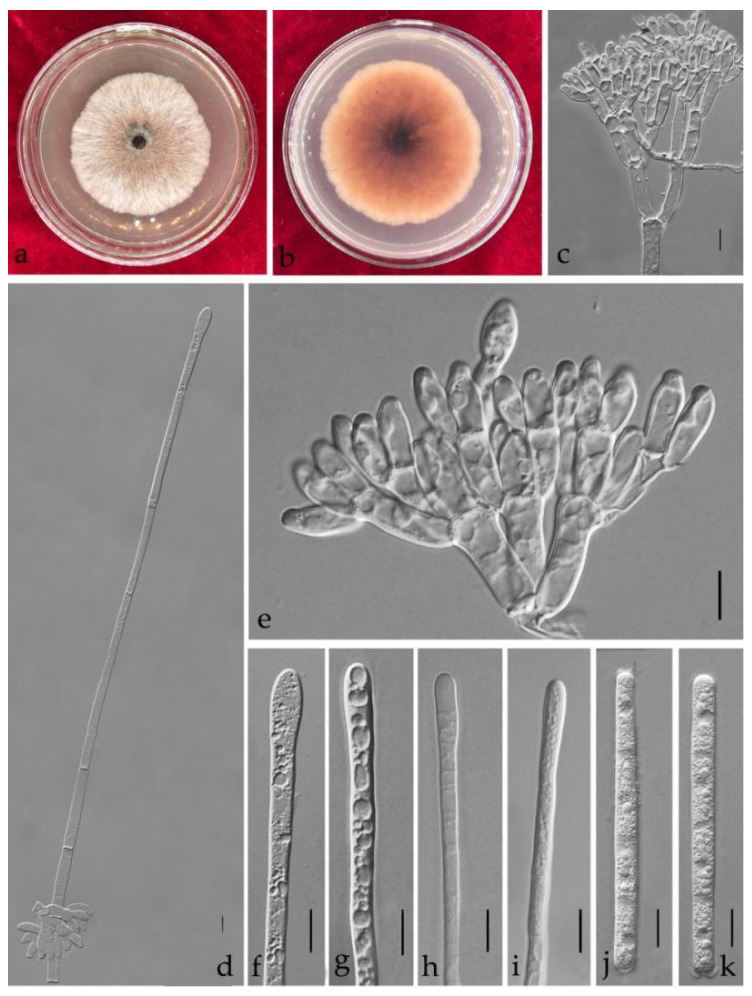
*Calonectria eucalypti* (ZHKUCC 21-0013: New host record) (**a**,**b**) Culture characteristics on MEA after 7 days (**a**) upper view; (**b**) reverse view); (**d**) Macroconidiophores; (**c**,**e**) Conidiogenous apparatus; (**f**–**i**) Vesicles; (**j**,**k**) Macroconidia; Scale bars: (**c**–**k**) = 10 μm.

**Figure 9:**
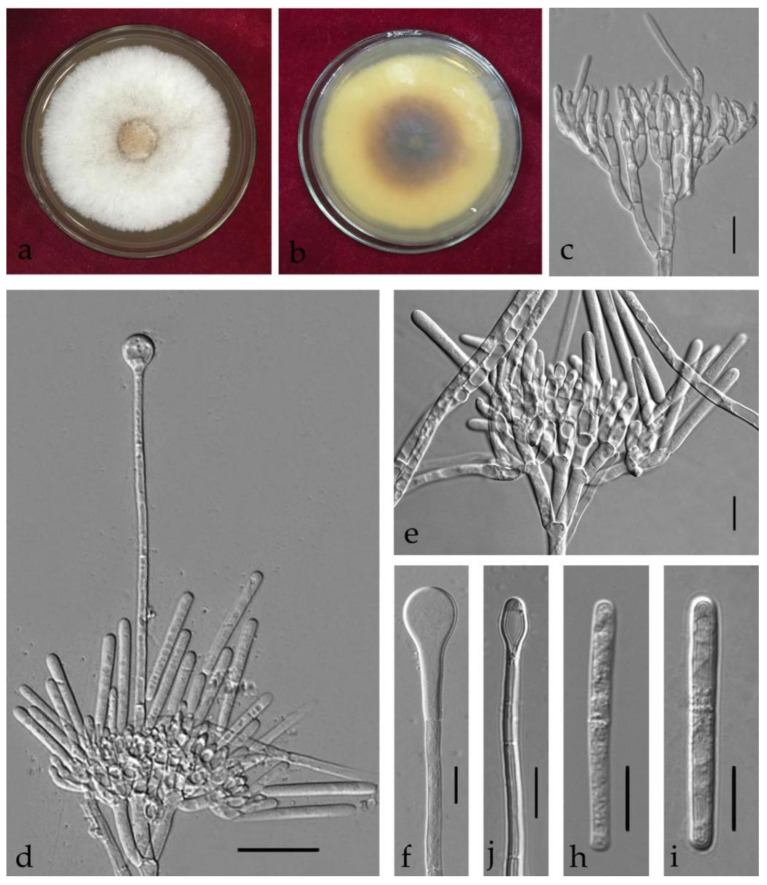
*Calonectria hongkongensis* (ZHKUCC 21-0016: New host record) (**a**,**b**) Culture characteristics on MEA after 7 days ((**a**) upper view; (**b**) reverse view); (**d**) Macroconidiophores; (**c**,**e**) Conidiogenous apparatus; (**f**–**j**) Vesicles; (**h**,**i**) Macroconidia; Scale bars: (**c**–**i**) = 10 μm.

**Table 1:** Primers information in this study.

Genes	Primers	Sequence 5′-3′	Annealing Temperature	References
*cmd*A	CAL-228F	GAGTTCAAGGAGGCCTTCTCCC	55 °C	[18]
	CAL-737R	CATCTTTCTGGCCATCATGG		
*tef*1-α	EF1-728F	CATCGAGAAGTTCGAGAAGG	54 °C	[18]
	EF2	GGARGTACCAGTSATCATGTT		[19]
β-tubulin	T1	AACATGCGTGAGATTGTAAGT	53 °C	[20]
	CYLTUB1R	AGTTGTCGGGACGGAAGAG		[21]

## Data Availability

All sequence data generated in this study is deposited in NCBI GenBank and accession numbers are given in the Supplementary Materials.

## Supplementary Materials

The following supporting information can be downloaded at: https://www.mdpi.com/article/10.3390/jof8070719/s1, Table S1. GeneBank accession numbers of Calonectria strains used in phylogenetic analysis.
