# Supplementary material for: Identification and Characterization of Calonectria Species Associated with Plant Diseases in Southern China
Source: J Fungi (Basel). 2022 Jul 9;8(7):719. doi: 10.3390/jof8070719 (PMC9324520; doi:10.3390/jof8070719)
Supplement: Supplementary file 1 [file jof-08-00719-s001.zip › jof-1807070-supplementary.pdf]

Journal of Fungi

MDPI

Supplementary Table S1. GeneBank accession numbers of *Calonectria* strains used in phylogenetic analysis. The isolates obtained in this study are bold.

Species	Culture collection number	GenBank accession number		
		*cmd*A	*tef*1-*α*	*β-tubulin*
Ca. acaciicola	CMW 47173^{T}	MT335160	MT412690	MT412930
Ca. acaciicola	CMW 47174	MT335161	MT412691	MT412931
Ca. acicola	CMW 30996^{T}	MT335162	MT412692	MT412932
Ca. acicola	CBS 114812	MT335163	MT412693	MT412933
Ca. aciculata	CERC 5342^{T}	MT335164	MT412694	MT412934
Ca. aconidialis	CMW 35174^{T}	MT335165	MT412695	N/A
Ca. aconidialis	CMW 31370	MT335167	MT412697	N/A
Ca. aconidialis	CMW 31390	MT335179	MT412709	N/A
Ca. aconidialis	CMW 31392	MT335169	MT412699	N/A
Ca. aconidialis	CMW 31439	MT335178	MT412708	N/A
Ca. aconidialis	CMW 31440	MT335177	MT412707	N/A
Ca. aconidialis	CMW 35169	MT335175	MT412705	N/A
Ca. aconidialis	CMW 35184	MT335174	MT412704	N/A
Ca. aconidialis	CMW 35187	MT335173	MT412703	N/A
Ca. aconidialis	CMW 35409	MT335171	MT412701	N/A
Ca. aconidialis	**ZHKUCC 210031**	**ON375352**	**ON375353**	**ON375354**
Ca. aeknauliensis	CMW 48253^{T}	MT335180	MT412710	N/A
Ca. aeknauliensis	CMW 48254	MT335181	MT412711	N/A
Ca. asiatica	CBS 114073^{T}	AY725741	AY725705	AY725616
Ca. australiensis	CMW 23669^{T}	MT335192	MT412723	MT412946
Ca. brasiliensis	CBS 133609	KC491222	KC491225	KC491228
Ca. brasiliensis	CBS 134818	KM395991	KM395817	KM395905
Ca. brasiliensis	CBS 230.515^{T}	MT335200	MT412731	MT412953
Ca. brasiliensis	CMW 32949	MT335201	MT412732	MT412954
Ca. brassicicola	CBS 112841^{T}	KX784561	KX784689	KX784619
Ca. bumicola	CMW 48257^{T}	MT335205	MT412736	N/A
Ca. canadiana	CMW 23673^{T}	MT335206	MT412737	MT412958
Ca. canadiana	CERC 8952	MT335290	MT412821	MT413035
Ca. auriculiformis	CMW 47178^{T}	MT335190	MT412721	MT412944
Ca. auriculiformis	CMW 47179	MT335191	MT412722	MT412945
Ca. auriculiformis	**ZHKUCC 210053**	**ON260793**	**ON260797**	**ON260801**
Ca. auriculiformis	**ZHKUCC 210054**	**ON260794**	**ON260798**	**ON260802**
Ca. cassiae	**ZHKUCC 210011^{T}**	**ON260790**	**MZ516860**	**MZ516863**
Ca. cassiae	**ZHKUCC 210012**	**ON260791**	**MZ516861**	**MZ516864**
Ca. cerciana	CMW 25309^{T}	MT335211	MT412742	MT412963
Ca. cerciana	CMW 25290	MT335212	MT412743	MT412964
Ca. chinensis	CMW 30986	MT335221	MT412752	MT412973

Supplementary Table S1. (Continued)

Species	Culture collection number	GeneBank accession number		
		*cmd*A	*tef*1-*α*	*β-tubulin*
Ca. chinensis	CMW 47192	MT335300	MT412831	MT413045
Ca. cochinchinensis	CMW 47187	MT335227	MT412758	MT412979
Ca. colhounii	CBS 293.79^{T}	GQ267373	GQ267301	DQ190564
Ca. colombiensis	CMW 23676^{T}	MT335228	MT412759	MT412980
Ca. colombiensis	CMW 30985	MT335229	MT412760	MT412981
Ca. crousiana	CMW 27249^{T}	MT335230	MT412761	MT412982
Ca. crousiana	CMW 27253	MT335231	MT412762	MT412983
Ca. curvispora	CMW 23693^{T}	MT335232	MT412763	N/A
Ca. curvispora	CMW 48245	MT335233	MT412764	N/A
Ca. cylindrospora	CBS 119670	MT335236	MT412767	MT412985
Ca. cylindrospora	CMW 30978	MT335237	MT412768	MT412986
Ca. cylindrospora	CBS 136425	MT335235	MT412766	MT412984
Ca. eucalypti	CMW 27209	MT335341	MT412872	MT413084
Ca. eucalypti	**ZHKUCC 210013**	**ON260792**	**MZ516862**	**MZ516865**
Ca. fujianensis	CMW 27254	MT335250	MT412781	MT412999
Ca. fujianensis	CMW 27257^{T}	MT335249	MT412780	MT412998
Ca. fujianensis	CBS 131802	MT335302	MT412833	MT413047
Ca.gordoniae	CMW 23694^{T}	MT335251	MT412782	MT413000
Ca. guangdongensis	**ZHKUCC 21-0062^{T}**	**MZ491127**	**MZ491149**	**MZ491171**
Ca. guangdongensis	**ZHKUCC 21-0063**	**MZ491128**	**MZ491150**	**MZ491172**
Ca. hawksworthii	CBS 111870^{T}	MT335254	MT412785	MT413003
Ca. hawksworthii	CMW 14878	MT335378	MT412909	MT413119
Ca. hawksworthii	CMW 31393	MT335247	MT412778	MT412996
Ca. heveicola	CMW 49913^{T}	MT335255	MT412786	MT413004
Ca. heveicola	CMW 49928	MT335280	MT412811	MT413025
Ca. heveicola	CMW 49935	MT335281	MT412812	MT413026
Ca. honghensis	CERC 5572^{T}	MT335256	MT412787	MT413005
Ca. honghensis	CERC 5571	MT335257	MT412788	MT413006
Ca. hongkongensis	CBS 114828^{T}	MT335258	MT412789	MT413007
Ca. hongkongensis	CERC 7132	MT335261	MT412792	MT413010
Ca. hongkongensis	**ZHKUCC 210016**	**MZ463742**	**MZ516838**	**MZ516851**
Ca. ilicicola	CMW30998^{T}	MT335266	MT412797	N/A
Ca. indonesiae	CMW23683^{T}	MT335267	MT412798	MT413015
Ca. indonesiae	CBS 112840	MT335268	MT412799	MT413016
Ca. insularis	CMW 30991^{T}	MT335269	MT412800	MT413017
Ca. insularis	CMW 30992	MT335270	MT412801	MT413018
Ca. kyotensis	CBS 114525^{T}	MT335271	MT412802	MT413019
Ca. kyotensis	CBS 114550	MT335246	MT412777	MT412995

Supplementary Table S1. (Continued)

Species	Culture collection number	GeneBank accession number		
		*cmd*A	*tef*1-*α*	*β-tubulin*
Ca. kyotensis	CBS 114692	MT335245	MT412776	MT412994
Ca. kyotensis	CERC 7126	MT335356	MT412887	MT413098
Ca. kyotensis	CMW 31411	MT335388	MT412919	MT413126
Ca. lageniformis	CBS 111324[T]	KX784574	KX784702	KX784632
Ca. lantauensis	CERC 3302[T]	MT335272	MT412803	N/A
Ca. lantauensis	CERC 3301	MT335273	MT412804	N/A
Ca. lateralis	CMW 31412[T]	MT335274	MT412805	MT413020
Ca. lichi	CERC 8866[T]	MT335278	MT412809	MT413023
Ca. lichi	CERC 8850	MT335279	MT412810	MT413024
Ca. lombardiana	CMW 30602[T]	MT335395	MT412926	MT413133
Ca. hawksworthii	CMW 14878	MT335378	MT412909	MT413119
Ca. maranhensis	CBS 134811[T]	KM396035	KM395861	KM395948
Ca. maranhensis	CBS 134812	KM396036	KM395862	KM395949
Ca. melaleucae	**ZHKUCC 21-0066[T]**	**MZ491110**	**MZ491132**	**MZ491154**
Ca. melaleucae	**ZHKUCC 21-0067**	**MZ491111**	**MZ491133**	**MZ491155**
Ca. monticola	CBS 140645[T]	KT964771	KT964773	KT964769
Ca. monticola	CPC 28836	KT964772	KT964774	KT964770
Ca. multiseptata	CMW 23692[T]	MT335299	MT412830	MT413044
Ca. pacifica	CMW 16726[T]	MT335311	MT412842	N/A
Ca. pacifica	CMW 30988	MT335312	MT412843	N/A
Ca. paracolhounii	CBS 114679[T]	KX784582	KX784714	KX784644
Ca. paracolhounii	CBS 114705	N/A	KX784715	KX784645
Ca.penicilloides	CBS 174.55	MT335338	MT412869	MT413081
Ca. plurilateralis	CBS 111401[T]	MT335340	MT412871	MT413083
Ca. propaginicola	CBS 134815[T]	KM396040	KM395866	KM395953
Ca. propaginicola	CBS 134816	KM396041	KM395867	KM395954
Ca. propaginicola	CBS 134824	KM396049	KM395875	KM395962
Ca. pseudoreteaudii	CMW 25310[T]	MT335354	MT412885	MT413096
Ca. pseudoreteaudii	CMW 25292	MT335355	MT412886	MT413097
Ca. pseudoreteaudii	CMW 31487	MT335348	MT412879	MT413090
Ca. pseudoreteaudii	CBS 133349	MT335352	MT412883	MT413094
Ca. queenslandica	CMW 30604[T]	MT335367	MT412898	MT413108
Ca. queenslandica	CMW 30603	MT335368	MT412899	MT413109
Ca. reteaudii	CMW 30984[T]	MT335370	MT412901	MT413111
Ca. reteaudii	CMW 16738	MT335371	MT412902	MT413112
Ca. shaoguanensis	**ZHKUCC 210036[T]**	**MZ491112**	**MZ491134**	**MZ491156**
Ca. shaoguanensis	**ZHKUCC 210037**	**MZ491113**	**MZ491135**	**MZ491157**
Ca. strelitziensis	**ZHKUCC 210019[T]**	**MZ491105**	**MZ491129**	**MZ491151**
Ca. strelitziensis	**ZHKUCC 210047**	**MZ491106**	**MZ491130**	**MZ491152**

Supplementary Table S1. (Continued)

Species	Culture accession	*GeneBank accession number*		
		*cmd*A	*tef*1-*α*	*β-tubulin*
Ca. strelitziensis	**ZHKUCC 210048**	**MZ491107**	**MZ491131**	**MZ491153**
Ca. sumatrensis	CMW 23698[T]	MT335382	MT412913	N/A
Ca. sumatrensis	CMW 30987	MT335383	MT412914	N/A
Ca. sumatrensis	CBS 112936	MT335380	MT412911	N/A
Ca. syzygiicola	CBS 112831[T]	N/A	KX784736	KX784663
Ca. tonkinensis	CMW 47430[T]	MT335384	MT412915	MT413122
Ca. uniseptata	CBS 413.67[T]	GQ267379	GQ267307	GQ267208
Ca. variabilis	CMW 3187[T]	MT335392	MT412923	MT413130
Ca. variabilis	CMW 2914	MT335393	MT412924	MT413131
Ca. yunnanensis	CERC 5339[T]	MT335396	MT412927	MT413134
Ca. yunnanensis	CERC 5337	MT335397	MT412928	MT413135
Ca. yunnanensis	CERC 5376	MT335361	MT412892	MT413103
Curvicladiella cignea	CBS 109167[T]	KM231287	KM231867	KM232002
Curvicladiella cignea	CBS 109168	KM231286	KM231868	KM232003

T: ex-type isolates of the *Calonectria* species. Sequences produced in this study are in bold. CBS: Westerdijk Fungal Biodiversity Institute, Utrecht, The Netherlands; CERC: China Eucalypt Research Centre, ZhanJiang, GuangDong Province, China; CMW: Culture collection of the Forestry and Agricultural Biotechnology Institute (FABI), University of Pretoria, Pretoria, South Africa; ZHKUCC, Zhongkai University of Agriculture and Engineering Culture Collection. N/A: information is not available.
